# Supplementary material for: Dual-Energy-CT for Osteitis and Fat Lesions in Axial Spondyloarthritis: How Feasible Is Low-Dose Scanning?
Source: Diagnostics (Basel). 2023 Feb 18;13(4):776. doi: 10.3390/diagnostics13040776 (PMC9955853; doi:10.3390/diagnostics13040776)
Supplement: Supplementary file 1 [file diagnostics-13-00776-s001.zip › diagnostics-2137568-supplementary.pdf]

diagnostics

MDPI

Supplement Table S1. Subpopulation analysis of patients without severe sclerosis.

**Osteitis (Patient level)**	**Overall**					**Reader 1** (Expert)	**Reader 2** (Beginner)
	**SE**	0%	**LR-**	1.33	**SE**	50%	0%
	**SP**	75%	**LR+**	0	**SP**	87.5%	87.5%
**Osteitis** (Joint level)							
	**SE**	11%	**LR-**	0.98	**SE**	22.22%	0%
	**SP**	90%	**LR+**	1.11	**SP**	95%	95%
**Fatty lesions** (Patient level)							
	**SE**	100%	**LR-**	0	**SE**	66.67%	66.67%
	**SP**	82.6%	**LR+**	5.75	**SP**	91.3%	60.87%
**Fatty lesions** (Joint level)							
	**SE**	50%	**LR-**	0.58	**SE**	50%	50%
	**SP**	85.9%	**LR+**	3.56	**SP**	92.98%	61.4%

Subpopulation analysis was performed in 26 patients and 69 joints. Two patients and nine joints were classified as positive for osteitis. Three patients and 12 joints showed fatty lesions. Diagnostic accuracy was not be improved by excluding patients with sclerosis. The expert reader achieved higher diagnostic accuracy for both lesions compared to the beginner.

Sensitivity (SE), specificity (SP), positive and negative likelihood ratio (LR+/LR-)

Supplement Table S2. Diagnostic perfomance seperated by gender.

		**SE**	**SP**	**LR-**	**LR+**
**Female**	Osteitis	60%	34,78%	1,15	0,92
	Fatty lesions	85,71%	66,67%	0,21	2,57
**Male**	Osteititis	80%	50%	0,4	1,6
	Fatty lesions	69,23%	66,67%	0,46	2,08

28 females and 40 males were included in the analysis. For osteitis, the diagnostic accuracy in women was lower compared to males. However, fatty bone marrow lesions were detected with higher sensitivity in female patients, while specificity was similar in both groups.

Sensitivity (SE), specificity (SP), positive and negative likelihood ratio (LR+/LR-)
